# Circulating tumor DNA: Opportunities and challenges for pharmacometric approaches

**DOI:** 10.3389/fphar.2022.1058220

**Published:** 2023-03-08

**Authors:** Benjamin Ribba, Andreas Roller, Hans-Joachim Helms, Martin Stern, Conrad Bleul

**Affiliations:** Roche Pharma Research and Early Development, Roche Innovation Center Basel, F. Hoffman-La Roche Ltd, Basel, Switzerland

**Keywords:** early clinical development, cancer immunotherapies, circulating tumor DNA (ctDNA), clinical efficacy, modeling and simulation

## Abstract

To support further development of model-informed drug development approaches leveraging circulating tumor DNA (ctDNA), we performed an exploratory analysis of the relationships between treatment-induced changes to ctDNA levels, clinical response and tumor size dynamics in patients with cancer treated with checkpoint inhibitors and targeted therapies. This analysis highlights opportunities for pharmacometrics approaches such as for optimizing sampling design strategies. It also highlights challenges related to the nature of the data and associated variability overall emphasizing the importance of mechanistic modeling studies of the underlying biology of ctDNA processes such as shedding, release and clearance and their relationships with tumor size dynamic and treatment effects.

## Introduction

Predicting long-term clinical benefit of anti-cancer drugs is notoriously difficult. Nevertheless, such predictions can play a key role in reducing the attrition rate of anti-cancer molecules in late phase clinical trials (Hutchinson and Kirk, 2011). Recently, circulating tumor DNA (deoxyribonucleic acid) or ctDNA, which can be collected longitudinally, has been shown to hold additional predictive power to imaging-based markers of response (Cescon et al., 2020). Pharmacometric (PMX) approaches can take the advantage of longitudinal measurements as demonstrated with tumor growth modeling approaches and, as such, represent an opportunity for ctDNA to inform new molecular entity (NME) clinical trial development with respect to identification of clinically most promising compounds, optimal sampling design, combination partners, and precision dosing.

**FIGURE 1:**
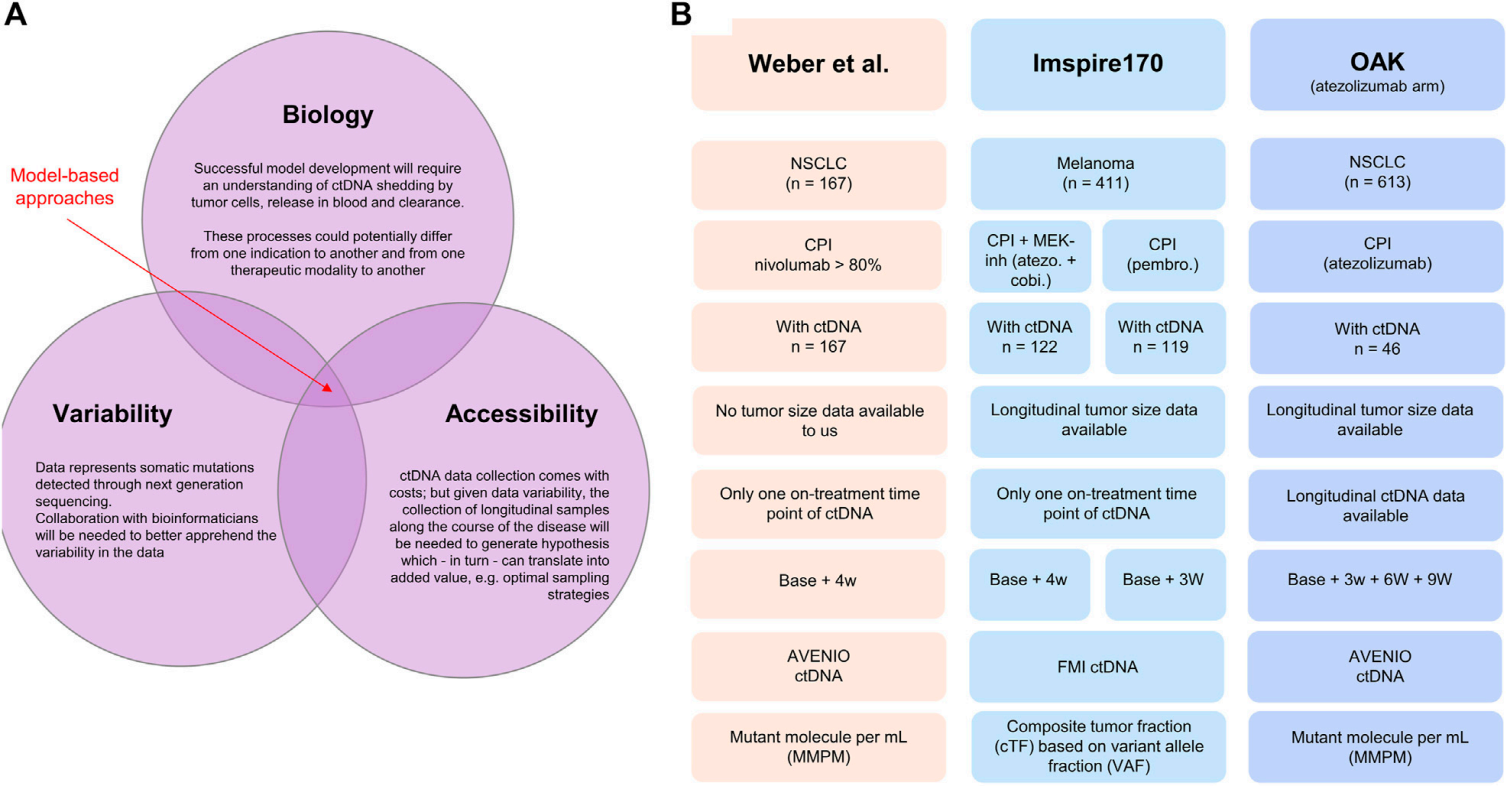
**(A)** Illustration of the interconnected challenges inherent to the successful development of a model-based approach for leveraging longitudinal ctDNA to support early clinical decision-making in oncology, namely, the access to highly longitudinal samples and the understanding of disease biology and bioinformatic nature of the data. **(B)** Database description: Weber et al. (2021) clinical study published in 2021. The dataset was shared with the authors without further obligations according to the policy of the Journal of Clinical Oncology Precision Oncology and according to the local patient-level sharing privacy rules. The dataset is composed of 167 patients with non-small cell lung cancer. The ctDNA sample collection was performed between 2015 and 2018. The Roche AVENIO assay was used for ctDNA measurements and reported as (average) mutant molecules per milliliter measured consistently at two time points (baseline, i.e., cycle 1 1 and 28 days in median, SD = 10 days). For 15 patients, the ctDNA value at both baseline and on treatment was recorded 0, making the calculation of change from baseline impossible. These patients were removed from the analysis. Patients suffered from stage IIIB (13.2%) or stage IV (86.8%) NSCLC; all were treated with checkpoint inhibitors (nivolumab for more than 80%). The overall response rate was 26.3% (*n* = 44), and the mean treatment duration was 13 cycles. Anonymized patient-level survival data were available. IMspire170: Roche sponsored study on patients with melanoma (Gogas et al., 2021) Data consisted of 411 patients, of which 209 patients were treated with the checkpoint inhibitor atezolizumab with the tyrosine kinase (MEK-) inhibitor cobimetinib and 202 patients with pembrolizumab. ctDNA measurements were reported as a ctDNA tumor fraction (cTF) based on aneuploidy and variant allele fraction (VAF) and were available for 241 patients (122 in the group treated with atezolizumab and cobimetinib and 119 in the group treated with pembrolizumab). Two time points were available: baseline (cycle 1 day 1) and cycle 2 day 1 (corresponding to 21 days for the pembrolizumab cohort and 28 days for the atezolizumab/cobimetinib cohort). Tumor size (sum of longest diameters) was available longitudinally for 409 patients out of the 411 patients. Patient-level survival data were available. OAK: Roche sponsored the study on non-small cell lung cancer patients treated with the checkpoint inhibitor atezolizumab or chemotherapy docetaxel (Rittmeyer et al., 2017). The study included 1,225 patients. ctDNA data were available for a subset only (*n* = 94). Our analysis included only the data from the atezolizumab arm (*n* = 613 and 46 with ctDNA data). The data from the chemotherapy arm were only used for developing the ctDNA time course model with the treatment arm regarded as a covariate. The Roche AVENIO assay was performed for ctDNA measurements and reported as mutant molecules per milliliter, and data at four time points were available: cycle 1 (baseline), cycle 2 (around 21 days), cycle 3 (around 42 days), and cycle 4 (around 63 days). These data were used for longitudinal modeling using the Stein et al. model. OAK ctDNA data analysis has been already published (Zou et al., 2021).

In this perspective, through the exploratory analysis of ctDNA data from nearly 500 cancer patients treated with checkpoint inhibitors and targeted therapies, we dissect the relationships between on-treatment ctDNA change over time from baseline and overall survival, clinical response, and tumor size dynamics. We believe this effort is a required first step for the further successful development of model-based approaches. The analysis also sheds light on interconnected challenges related to the specific nature of the data, associated variability, and complexity of underlying biology of ctDNA processes such as shedding, release, and clearance (Avanzini et al., 2020) and their relationships with tumor size dynamics and treatment effects (see Figure 1A).

## Problem statement

Traditionally, the rate of “best overall response” gives an indication of patients’ early response to treatment based on repeatedly quantifying the size of one or several cancer lesions by radiographic imaging. The overall response rate (ORR) is the percentage of patients achieving a complete or partial response through RECIST 1.1 at any time of the treatment (Ruchalski et al., 2021). Observing a high ORR in early clinical studies is encouraging and often ungates further studies and investment. A low ORR, on the contrary, could amongst others, indicate an absence of efficacy and can support the decision to stop development of an experimental treatment. However, the correlation between ORR and long-term clinical benefit is limited, in particular for cancer immunotherapy which does not act directly by killing tumor cells but rather stimulates an anti-tumor response (Gerwing et al., 2019; Goulart et al., 2022). For tebentafusp, a T-cell bispecific cancer immunotherapy approved recently for the treatment of metastatic uveal melanoma, approval was based on the observed improvement of overall survival in a randomized phase III trial. Early clinical trials had shown many patients remaining on trial for a long time in stable disease, however, with a radiological response rate (RR) of only 12% (Carvajal et al., 2022). Such a low response rate could have led many drug developers to stop the development of what is, in reality, an efficacious medicine. This example indicates that while RR could be associated with high specificity for identification of drugs that convey a survival benefit, sensitivity might be low. As a consequence, complementing RR information with data that can hold predictive potential is key for decision-making in oncology development where decisions to invest in large and costly confirmatory clinical trials typically relies on the results of previously conducted clinical studies (phase I - II) with a limited number of patients (Cannarile et al., 2021).

The PMX community has been, for many years, contributing to the question of how to improve early decision-making. It proposes to leverage the time course of tumor size instead of relying on a categorical score (RECIST 1.1) derived from the comparison of the sum of the longest diameters (SLD) between baseline from up to five measurable target lesions and one given time point (the one at which the best response is observed) (Yates and Cheung, 2021). As such, PMX approaches are ideally positioned to integrate informative data collected longitudinally. In fact, efforts to complement the existing state-of-the-art tumor size kinetic models with other relevant biomarkers have been an important area of research for many years (Netterberg et al., 2020).

Liquid biopsies enabling the measurement of ctDNA have emerged as a promising technology to overcome some of the limitations discussed previously (Zhang et al., 2020). When tumor cells die from apoptosis or necrosis, their DNA is shed and released into circulating blood (Heitzer et al., 2020). Technologies such as next-generation sequencing (NGS) can detect somatic mutations and quantify ctDNA in terms of variant allele frequency (VAF, ratio between the number of mutated- and wild-type DNA copies) or mutant tumor molecules per milliliter of plasma (MMPM) (Bos et al., 2021). Numerous studies have now been published on the potential of ctDNA for screening or for characterizing disease biology (Cheng et al., 2016). Another application with a lot of potential with respect to the opinions discussed previously is the use of ctDNA as a measure of disease burden and with this, as a predictor of long-term clinical benefit (Bratman et al., 2020). In the aforementioned example of tebentafusp, ctDNA change from baseline within 9 weeks following treatment start was associated with long-term clinical benefit. It was also reported that the degree of ctDNA reduction correlated with overall survival and that this association was largely independent from the radiological response categorization (Shoushtari et al., 2021).

## Circulating tumor DNA and overall survival

To better understand the potential of model-based approaches to explore ctDNA data, we built a dataset composed of 454 patients from three published clinical studies (Rittmeyer et al., 2017; Gogas et al., 2021; Weber et al., 2021). Two studies focused on non-small cell lung cancer (*n* = 213 patients in total: 167 in one study and 46 in another) and one on melanoma (*n* = 241). The studies involved different types of treatment: immunotherapy with a checkpoint inhibitor (CPI: atezolizumab, nivolumab, pembrolizumab, durvalumab, and ipilimumab) alone (*n* = 332, pooling data from three studies) or in combination with targeted therapy (atezolizumab + cobimetinib) (*n* = 122, from one study). Indication, treatment, and sampling design of ctDNA and technology for its quantification were different between the studies. A summary of the analyzed dataset is illustrated in Figure 1B.

**FIGURE 2:**
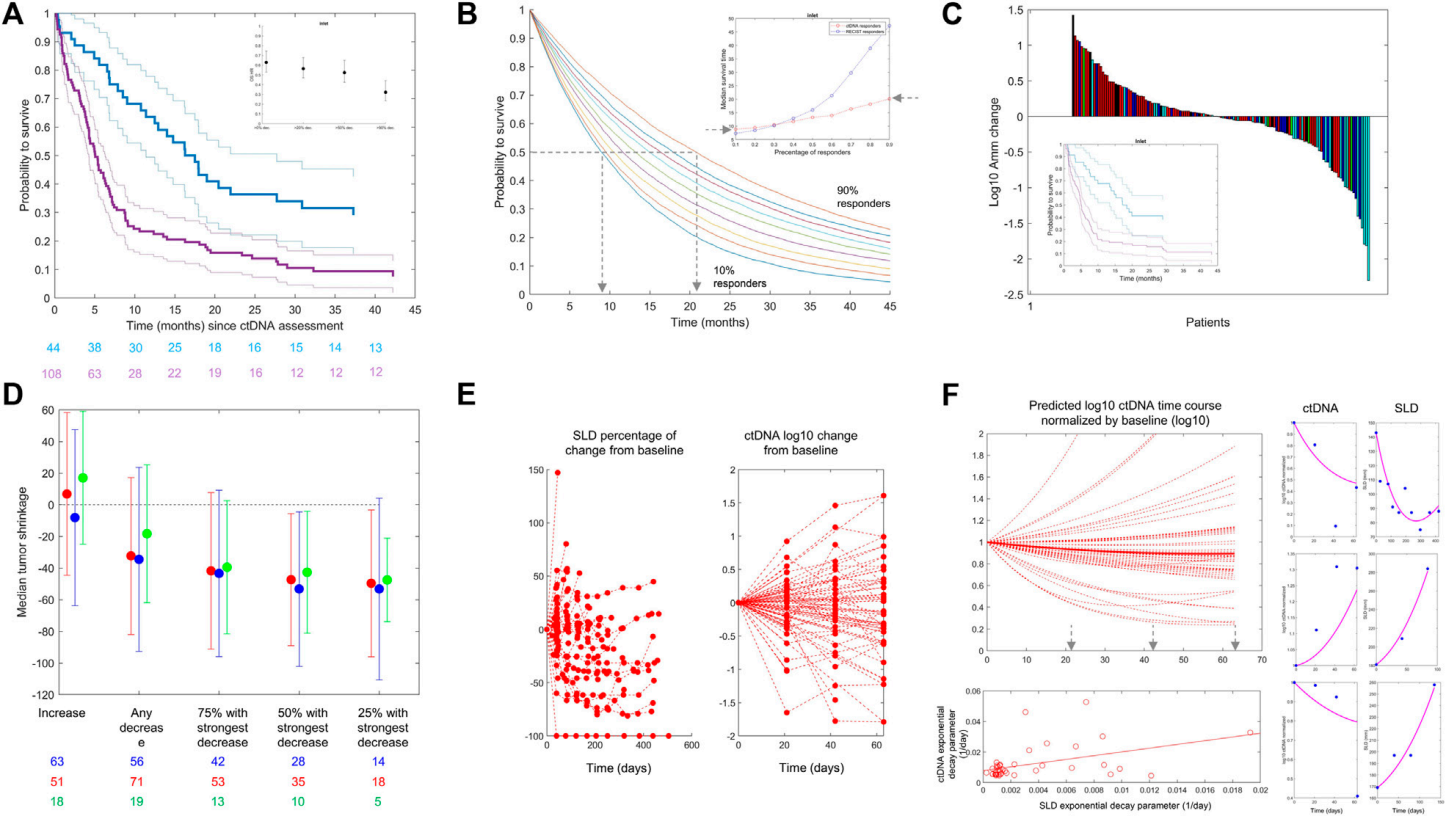
**(A)** From the Weber et al. dataset. Kaplan–Meier estimates of overall survival stratified by ctDNA change on treatment *versus* baseline: decrease >50% (blue line) *versus* all other patients (purple line). Confidence intervals are shown with thin lines. A: Data from Weber et al., NSCLC patients treated with CPI. The inset graphic shows the evolution of OS HR when reducing the ctDNA decrease cut-off (from left to right). The chosen cut-off values (20, 50, and 90% ctDNA reduction) represent approximately the observed quartiles of ctDNA reductions. More reduction is associated with better HR. **(B)** Kaplan–Meier (KM) curves are simulated using the exponential distribution parameterized according to the observed hazard rates of responders in terms of ctDNA drop from the Weber et al. dataset. Each colored line is a KM curve for a given percentage of responders from 10 to 90%. The inset shows the evolution of the median survival time (time at which half of the population is still alive) as a function of the percentage of responders for both ctDNA (red) and RECIST 1.1 (blue). **(C)** From the Weber et al. dataset. Waterfall plots of ctDNA colored by best overall response following RECIST 1.1 categorization. Complete response (green), partial response (cyan), stable disease (blue), and progressive disease (red) are shown. The inset shows Kaplan–Meier curves using ctDNA decrease less (purple) or more (blue) than 50% (like in panel **(A)**) in the patients within the same RECIST 1.1 category “stable disease.” **(D)** Median tumor change (*y*-axis) in patient subgroups defined by the ctDNA decrease cut-off (*x*-axis) for atezolizumab + cobimetinib (red) and pembrolizumab (blue) for patients with melanoma in IMspire170 and atezolizumab for patients with NSCLC in OAK (green). The length of the error bars corresponds to ± 1 SD. **(E)** OAK data: Tumor size (left) and ctDNA (right) change from baseline over time. **(F)** Left/top: Model-based predictions of ctDNA time course in patients with NSCLC treated with atezolizumab (OAK study) using the Stein et al. model, omitting the baseline term and using individual parameter estimates. Left/bottom: Scatter plot of predicted exponential decay for ctDNA *versus* same parameter for tumor size (dots) (see equation 1). The line is a least square regression. Right: Individual fits for the joined ctDNA–SLD model proposed in equation (2). The model can fit data when ctDNA and SLD time courses are positively correlated (top) or negatively correlated (bottom). We here report individual fits as illustration only. The model can fit different types of profiles although parameters of the model were not estimated with sufficient precision to use this model for any predictive purpose.

Based on the data from Weber et al. (2021), which were shared with us without further obligations according to the policy of the Journal of Clinical Oncology Precision Oncology, we show in Figure 2A Kaplan–Meier curves of patients’ overall survival as a function of early change in ctDNA. We selected the commonly used 50% ctDNA reduction from baseline as the cut-off (Nabet et al., 2020; Weber et al., 2021; Zou et al., 2021). The two curves separate, and the patients who achieve a 50% ctDNA reduction have a larger probability to live longer than those who do not. We calculated hazard ratios using varying cut-offs of ctDNA change from baseline and showed that a higher degree of ctDNA decrease is associated with a lower hazard ratio (i.e., longer survival), similar to what has been reported for tebentafusp (Figure 2A, inset).

To further evaluate the impact of ctDNA dynamics on the long-term clinical outcome (e.g., overall survival), we used the same dataset to perform a simulation study using RECIST 1.1 response criteria as a comparator. For RECIST, we defined response as complete response (CR) or partial response (PR). For ctDNA, we kept the commonly used threshold of 50% drop at week 4 from baseline. We generated overall survival data using exponential distribution parameterized with the observed hazard rates and performed simulations with a virtual population of 10,000 patients, varying the percentage of responders in the population from 10 to 90%, plotted corresponding Kaplan–Meier curves, and derived median survival time as a function of percentage of response for both ctDNA and RECIST 1.1 (Figure 2B). Finally, we selected a landmark of 6 months and calculated survival at this time point. We found that with 10% of responders in terms of ctDNA, 62% of patients would be alive at 6 months (56% when response is defined by RECIST 1.1), while 90% of responders would translate into a survival for 81% of patients at the landmark (89% with RECIST 1.1). From these data, we evaluate that 10% more ctDNA responders would translate into 1–2 months of survival benefit. It is interesting to note that the increase in the median survival time as a function of the percentage of responders is greater with RECIST 1.1 than it is for ctDNA (Figure 2B, inset).

To evaluate if ctDNA holds predictivity independent of RECIST 1.1, we performed a multivariate Cox proportional hazard model regression. Hazard ratios (HRs) were estimated to be 0.12 and 0.6 for RECIST 1.1 and ctDNA, respectively. The lower HR obtained for RECIST 1.1 is consistent with what is observed in the inset of Figure 2B. In this model, no dependency could be detected supporting the hypothesis of the independent predictive value of ctDNA. In addition to that, the difference in the time of assessment of RECIST 1.1 and ctDNA could contribute to the difference in the parameter estimates. Best overall response (BOR) by RECIST 1.1 can be taken at any time with first tumor size assessment typically occurring at week 6, while ctDNA data were taken at week 4 in this dataset. Overall, these findings are in line with the high sensitivity/low specificity of RECIST 1.1-based criteria as discussed previously.

## Circulating tumor DNA and clinical response

We further looked into potential relationships between ctDNA change and overall response with waterfall plots of ctDNA change colored by BOR (Figure 2C). Given the presence of large variation in the data, it is common to represent ctDNA change from baseline in terms of (base 10) logarithmic change. We see a clustering of best responses (lighter colors) within the negative ctDNA change from baseline, i.e., reduction. The rate of RECIST 1.1 responders (complete or partial response) was 35% (28/79) in patients with ctDNA decrease from baseline. Among the 73 patients with ctDNA increase, less patients were RECIST 1.1 responders (22%; 16/73). This clustering was observed consistently in the two other datasets. For patients treated with OAK (atezolizumab arm), 37% (7/19) of patients with ctDNA decrease were RECIST 1.1 responders, compared to only 11% (2/18) for patients with ctDNA increase. In IMspire170 (both arms together), 53% (125/238) of the patients experienced a decrease in ctDNA. Among them, 52% (65/125) had a complete or partial response compared to 32% (36/113) of patients who had ctDNA increased or unchanged.

Also, similar to tebentafusp and other reported data, ctDNA provides more granularity than overall response assessed through RECIST 1.1 because in patients from the same response categories (stable disease), a cut-off of ctDNA change can still separate patients in terms of survival benefit (inset of Figure 2C).

## Circulating tumor DNA and tumor size change

The previous analysis was further extended by looking into potential relationships between ctDNA change and quantitative tumor size change (rather than the RECIST 1.1 response category) in the studies where longitudinal tumor size data were available to us (i.e., OAK and IMspire170). We calculated the maximal change in the sum of the longest diameters each patient experienced across the whole time window of tumor size data collection. This value can be negative (in case of tumor size shrinkage) or positive (in case of tumor size increase). We show in Figure 2D the result of the analysis for IMspire170 (atezolizumab + cobimetinib (red) and pembrolizumab (blue)) and OAK atezolizumab datasets (green points). There was a relationship between the magnitude of ctDNA decrease (*x*-axis) taken at a fixed time point and the maximum tumor size change. The stronger the early ctDNA drop, the more the tumor shrinkage observed.

To try to elucidate the relationships between ctDNA data and tumor size, we jointly looked at the SLD and ctDNA time courses in the OAK dataset for which we had several time point measurements of ctDNA. Consistently at all cycles, we found that the majority of patients with ctDNA decrease have an early (week 6) tumor size decrease: 50% [(11/22) at cycle 2 (week 3), 63% (12/19) at cycle 3 (week 6), and 65% (11/17) at cycle 4 (week 9)]. The majority of patients with ctDNA increase have an early tumor size increase [62% (13/21), 65% (11/17), and 64% (9/14) at cycles 2, 3, and 4, respectively], altogether suggesting a link between early ctDNA and tumor size change.

## Optimal sampling strategy and the challenge of variability

Circulating tumor DNA data collection and quantification come at a significant cost (burden for patients, for study operations, and financially). Therefore, designing methods for optimal sampling of ctDNA is important.

We used an empirical approach where we modeled the ctDNA time course using a bi-exponential model classically used to capture tumor size dynamics, also called the Stein model (Stein et al., 2011):

$$y=y_{0}\cdot \left(\exp\left(-k_{s}\cdot t\right)+\exp\left(k_{g}\cdot t\right)-1\right),$$

where $k_{s}$ is the decay parameter, $k_{g}$ is the regrowth parameter, and $y_{0}$ is the initial value which was not estimated but fixed to the observed baseline value.

All ctDNA data were expressed in terms of (average) mutant molecule per ml (MMPM) and log(base 10)-transformed. Monolix (version 2021 R1, Lixoft SAS, a Simulation Plus company) was used to estimate the two parameters of the structural model and parameter(s) of a constant error model within a population framework, allowing to estimate variability in these parameters in the population. All population parameters were estimated with a low or moderate standard error [RSE]: 30.1% and 27.4% for the growth and decay rate fixed effect, respectively; 25% and 20% for the inter-individual variability random effects; and less than 5% for the residual error model parameter.

With this model, we reproduced the predicted time course of ctDNA in patients treated with atezolizumab (Figure 2F, top left). For patients with a decrease in ctDNA, we identified the time at which the ctDNA time course achieved its nadir following the idea that this would be the theoretical time at which the ctDNA measures hold the highest information. The result of this analysis shows that 21 days or cycle 2 might be too early for informative ctDNA measurements as the majority of the simulated patients had their ctDNA nadir beyond cycle 4, i.e., 9 weeks.

However, large inter-individual variability parameters were estimated, specifically for both decay and growth rates with an estimate close to 100% (assessed through the standard deviation of the random effects). These parameters were estimated with reasonable precision, which overall indicates the high degree of inter- and intra-individual variability (see Appendix for further details).

It is important to highlight that the nature of the data themselves, being a summarized statistics based on a number of alleles which most likely change from one time point to the other (not the same allele will contribute to the final data readout), may have a large contribution to the observed time course variability. Given the identified variability, the collection of longitudinal sampling of ctDNA along the course of the disease is needed to generate hypotheses on the optimal sampling strategy. This also appears important given that optimal strategies should not be “one size fits all”—as different drugs and mechanisms of action could be associated with different optimal sampling times.

## Joint modeling of ctDNA and tumor size time course and the challenge of biological complexity

When we applied the model by Stein et al. (Stein et al., 2011) to the SLD time course, which was independent of the ctDNA data, we found that the resulting parameter governing the decay slightly correlated with the same parameter for ctDNA (Figure 2F bottom left) (r = 0.45), supporting the hypothesis of the presence of a mechanical link between ctDNA and tumor size.

To follow up, we tested a simple joint model of ctDNA and SLD time course encoding the correlation between the two decay phenomena:

$$SLD\left(t\right)=SLD_{0}\cdot \left(\exp\left(-k_{sT}\cdot t\right)+\exp\left(k_{gT}\cdot t\right)-1\right),$$

$$ctDNA\left(t\right)=ctDNA_{0}\cdot \left(\exp\left(-\zeta \cdot k_{sT}\cdot t\right)+\exp\left(k_{g}\cdot t\right)-1\right),$$

where SLD denotes the sum of the longest diameter and $SLD_{0}$ its baseline value. $k_{gT}$ is the SLD growth parameter and $k_{sT}$ is the decay rate which we found again in the equation for ctDNA. The parameter ζ links the time course dynamics of SLD and ctDNA data. For data fitting, the population parameters were all fixed to values obtained when fitting ctDNA and SLD data independently (see Appendix for further details). Only the parameter ζ and its variability were estimated. Consistent with the hypothesis of the mechanical link between the two observations, its population value was 1.94 (RSE of 37%) and variability was 0.86 (RSE of 35%).

This model could reproduce patients’ ctDNA and SLD data through its flexibility to capture both expected (Figure 2F, right side top and middle row) and unexpected profiles (bottom row), i.e., ctDNA increase and SLD decrease, or the contrary. We believe that the ability of such a model to reproduce patient-level data is an additional support to the hypothesis of a link between ctDNA and SLD time course in the case of the checkpoint inhibitor and should be viewed as an incentive for the development of further modeling attempts.

However, proper joint modeling of ctDNA and SLD time course will require an understanding of ctDNA shedding by tumor cells, ctDNA release into blood, and clearance. These processes could potentially differ from one indication to another and from one therapeutic modality to another. Some mechanistic modeling efforts have been already undertaken. For example, Avanzini et al. (2020) modeled the mechanisms of ctDNA shedding and release into circulation; they assumed that ctDNA is shed by dying tumor cells with a certain shedding probability and that the half-life of ctDNA in circulation is around 30 min.

## Conclusion

In many recent studies, ctDNA has shown potential to be used as a powerful biomarker for long-term clinical benefit of patients receiving anti-cancer treatment. ctDNA has obvious advantages over other techniques such as imaging; it is less invasive and time-consuming and can theoretically provide a high quality estimate of the total tumor burden, while imaging-based techniques focus on a limited number of identified target lesions. Moreover, it could become less costly as technology evolves.

From this perspective, we have compiled a large dataset of patients (∼500 patients) with varying tumor indications (non-small-cell lung carcinoma (NSCLC) and melanoma), treatments (CPI ± targeted therapy) and ctDNA panels (AVENIO and FMI), and sampling times (longitudinal and static).

Our objective was neither to provide a holistic introduction to ctDNA and associated technology nor to showcase a sophisticated ctDNA data modeling framework. Clearly, much work remains to be conducted to improve such models and what we reported should serve as an introduction to this problem. Rather, through statistical and empirical modeling of this dataset, we wish to contribute to familiarize the PMX community with the opportunity that ctDNA modeling can represent the early clinical development of anti-cancer therapeutic agents and raise awareness of potential challenges.

The presented analysis also sheds some light on the challenges to be expected when it comes to using ctDNA data for decision-making. First, we need to consider the nature of the data and their associated variability: coming from bioinformatic readout, modelers will need to closely work with bioinformaticians to effectively model the data. Then, the accessibility to longitudinal data should be taken into account notwithstanding the cost of these measurements. Longitudinal assessments should offer the opportunity of a better understanding given the potentially high level of variability in the data. This should lead to more precise mathematical formulation of the underlying biological processes which contributes to increase the quality of modeling readouts, reduces variability, and in turn contributes to optimal sampling strategies given an underlying hypothesis between ctDNA shedding/release, treatment action, and tumor dynamics.

In conclusion, our analysis, in agreement with published literature, makes ctDNA an ideal candidate—based on its predictive potential—for integration within a pharmacometric framework to complement the current state-of-the-art tumor growth kinetic models.

By focusing efforts on promoting collection of longitudinal data, understanding of underlying biology and the nature of the data and their variability, there is hope for the development of successful modeling frameworks jointly describing ctDNA, tumor size, and long-term clinical benefit, which overall can significantly contribute to answering key questions around early identification of most promising compounds and precision dosing.

## Data Availability

The data analyzed in this study is subjected to the following licenses/restrictions: summary data have been already published. Raw data are not public. Requests to access these datasets should be directed to Weber, S., et al., Dynamic Changes of Circulating Tumor DNA Predict Clinical Outcome in Patients With Advanced Non-Small-Cell Lung Cancer Treated With Immune Checkpoint Inhibitors. JCO Precis Oncol, 2021. 5: p. 1540-1553; Zou, W., et al., ctDNA Predicts Overall Survival in Patients With NSCLC Treated With PD-L1 Blockade or With Chemotherapy. JCO Precis Oncol, 2021. 5: p. 827-838; Gogas, H., et al., Cobimetinib plus atezolizumab in BRAF(V600) wild-type melanoma: primary results from the randomized phase III IMspire170 study. Ann Oncol, 2021. 323): p. 384-394.
