# Supplementary material for: Circulating tumor DNA: Opportunities and challenges for pharmacometric approaches
Source: Front Pharmacol. 2023 Mar 8;13:1058220. doi: 10.3389/fphar.2022.1058220 (PMC10030934; doi:10.3389/fphar.2022.1058220)
Supplement: Supplementary file 1 [file DataSheet1.pdf]

Supplementary Material

1 Supplementary Data

This appendix provides more details on the longitudinal modeling of tumor size and ctDNA data as reported in panel F of Figure 2.

The ctDNA data were first modeled independently of the tumor size data using the model by Stein et al. [1]. For this, the ctDNA data (average MMPM) were logarithmically transformed (base 10). The baseline value was used as a regressor (not estimated). The regression problem was formulated using a population approach where the 2 (population) parameters (or fixed effects) were associated to random effects.

$$y_{ij} = f(t_{ij}; \beta, \eta_i) + e_{ij}, \qquad 1 \le i \le N, 1 \le j \le n_i$$

Where N denotes the number of individuals and n_i the number of data points of individual i.

β and η_i are the 2x1 vectors of fixed and individual effects. ε_{ij} denote the residual errors and are expressed as follows:

$$e_{ij} = g(t_{ij}; \beta, \eta_i) \cdot \varepsilon_{ij}, \qquad 1 \le i \le N, 1 \le j \le n_i$$

Where ε_{ij} is a random variable with mean 0 and variance 1 for identifiability reasons.

$$\varepsilon_{ij} \underset{i.i.d}{\sim} \mathcal{N}(0,1) \quad 1 \leq i \leq N, 1 \leq j \leq n_i$$

g, the (residual) error model, can take many forms. In our analysis, we have used constant error model: $g(t_{ij}; \beta, \eta_i) = a_i$, and combined error model $g(t_{ij}; \beta, \eta_i) = a_i + b_i \cdot f(t_{ij}; \beta, \eta_i)$.

The vector of population parameters β is composed by two parameters: $k_{g_{\text{pop}}}$ and $k_{s_{\text{pop}}}$.

Individual parameters, k_{g_i} and k_{s_i} were assumed to be log-normally distributed:

$$\log(k_{g_i}) \sim \mathcal{N}\left(\log\left(k_{g_{\text{pop}}}\right), \omega_{k_g}^2\right)$$

$$\log(k_{s_i}) \sim \mathcal{N}\left(\log\left(k_{s_{\text{pop}}}\right), \omega_{k_s}^2\right)$$

We assumed no correlation between the random effects associated to the two parameters.

We report below the parameter estimates and precision as relative standard error (RSE) in percent.

**Name**	**Description (unit)**	**Value**	**R.S.E (%)**
**Fixed effects**			
$k_{g_{\text{pop}}}$	ctDNA growth rate (1/day)	0.0038	30.1
$k_{s_{\text{pop}}}$	ctDNA decay rate (1/day)	0.0081	27.4
**Standard deviation of the random effects**			
ω_{k_g}	-	0.82	25.0
ω_{k_s}	-	1.02	19.9
**Error model parameters**			
a	Constant error model (m)	0.27	4.8

The simulation of ctDNA time course in panel F was done through simulating the model by Stein et al. [1] using the estimated individual parameter (maximum a posteriori) for all 46 patients and omitting the baseline value, explaining why all curves start at value 1.

The tumor size data (sum of the longest diameters) were first modeled independently of the ctDNA data using the same model and following the same process as described above. The result of parameter estimation is presented below:

**Name**	**Description (unit)**	**Value**	**R.S.E (%)**
**Fixed effects**			
$k_{gT_{\mathrm{pop}}}$	SLD growth rate (1/day)	0.0016	14.3
$k_{sT_{\mathrm{pop}}}$	SLD decay rate (1/day)	0.0014	29.0
**Standard deviation of the random effects**			
$\omega_{k_{gT}}$	-	1.03	10.7
$\omega_{k_{sT}}$	-	1.64	15.4
**Error model parameters**			
a	Constant part of combined error model (mm)	0.65	28.1
b	Proportional part of the combined error model	0.08	7.1

The best model for the residual errors was a combined model (additive and proportional). Note that these results were obtained by analyzing simultaneously not only the patients treated with atezolizumab but also the patients treated with docetaxel (the other arm of the study). The arm was

treated as a categorical covariate enabling the use of the individual parameters only for the atezolizumab arm (panel F, bottom left, x-axis).

For the joint ctDNA and SLD model, all population parameters (fixed and random effects) were fixed to the values reported above. Only the interaction parameter and its variability were estimated as well as the parameters of the two error models (assumed constant). The result of parameter estimation is presented below:

**Name**	**Description (unit)**	**Value**	**R.S.E (%)**
**Fixed effects**			
ζ_{pop}	Link between SLD and ctDNA decay rate	1.94	37.3
**Standard deviation of the random effects**			
ω_{ζ}	-	0.86	35.0
**Error model parameters**			
a (ctDNA)	-	0.024	6.25
a (SLD)	mm	7.91	5.07

1. Stein, W.D., et al., *Tumor regression and growth rates determined in five intramural NCI prostate cancer trials: the growth rate constant as an indicator of therapeutic efficacy.* Clin Cancer Res, 2011. **17**(4): p. 907-17.
